# *Mirage de tuberculose* in the 21^st^ century

**DOI:** 10.5588/pha.24.0056

**Published:** 2024-06-01

**Authors:** M.B. Kaelin, S. Wieser, B. Preiswerk, P.W. Schreiber, D. Russenberger, P. Kaiser, B. Schulthess, J. Nemeth

**Affiliations:** ^1^ Department of Infectious Diseases and Hospital Hygiene, University Hospital Zurich, University of Zurich, Zurich,; ^2^ Lungen-Klinik Bethanien, Zürich,; 3 Department of Infectious Diseases, Stadtspital Zürich Triemli, Zürich,; 4 Department of Infectious Diseases, Luzerner Kantonspital, Lucerne,; 5 Institute of Medical Microbiology, University of Zurich, Zurich, Switzerland

**Keywords:** transient culture positivity, public health perspective, drug-resistant TB

## Abstract

The occurrence of transient culture positivity for *Mycobacterium tuberculosis* (MTB), known as *mirage de tuberculose*, poses significant challenges in understanding its spectrum and implications. Here, we report a case of transient culture positivity, oscillating between detectable and non-detectable MTB cultures with minimal radiological features and review the literature on this phenomenon. The scarcity of scientific literature on this subject stems from the inherent impossibility of systematically studying mirage de tuberculose. Ethical and public health concerns prevent withholding treatment to monitor spontaneous reversion to negative cultures. Based on the literature, we estimate that mirage de tuberculose occurs in approximately one-third of individuals infected with MTB who exhibit no symptoms. Despite the inherently limited nature of these findings, they suggest that the significance of mirage de tuberculose may be greater than currently perceived. Managing cases of mirage de tuberculose presents formidable challenges from a public health perspective. Striking a balance between prompt treatment initiation to prevent transmission and the risk of unnecessary treatment requires careful consideration. In conclusion, mirage de tuberculose remains a poorly understood clinical entity with very limited literature available. Advancing research and interdisciplinary collaborations are essential to unravel the intricacies of this phenomenon and develop effective strategies to address its public health challenges.

## Abstract (fr)

L'apparition d'une culture transitoire positive pour *Mycobacterium tuberculosis* (MTB), connue sous le nom de mirage de tuberculose, pose des défis importants dans la compréhension de son spectre et de ses implications. Nous rapportons ici un cas de positivité transitoire des cultures, oscillant entre des cultures MTB détectables et non détectables avec des caractéristiques radiologiques minimales et passons en revue la littérature sur ce phénomène. La rareté de la littérature scientifique sur ce sujet provient de l'impossibilité inhérente d'étudier systématiquement le mirage de tuberculose. Des préoccupations éthiques et de santé publique empêchent l'interruption du traitement pour surveiller le retour spontané à des cultures négatives. Sur la base de la littérature, nous estimons que le mirage de tuberculose survient chez environ un tiers des personnes infectées par le MTB qui ne présentent aucun symptôme. Malgré la nature intrinsèquement limitée de ces résultats, ils suggèrent que l'importance du mirage de tuberculose pourrait être plus grande que ce que l'on perçoit actuellement. La prise en charge des mirages de tuberculose présente des défis considérables du point de vue de la santé publique. Il faut trouver un équilibre entre l'instauration rapide du traitement pour prévenir la transmission et le risque d'un traitement inutile. En conclusion, le mirage de tuberculose reste une entité clinique mal comprise et la littérature disponible est très limitée. L'avancement de la recherche et les collaborations interdisciplinaires sont essentiels pour démêler les subtilités de ce phénomène et élaborer des stratégies efficaces pour relever ses défis en matière de santé publique.

Within the realm of infectious diseases, the complexities surrounding *Mycobacterium tuberculosis* (MTB) infection remains a significant challenge for healthcare personnel. Caused by MTB, TB presents a wide spectrum of clinical manifestations, ranging from asymptomatic infection to severe disseminated disease. This intricate interplay between pathogen and host gives rise to what has been described as a spectrum of disease in the field of MTB research, wherein individuals may traverse different stages or manifestations over time.^1–3^

Interestingly, most people who harbour detectable MTB do not exhibit any symptoms of active TB disease. Instead, they reside within the spectrum of asymptomatic infection, a state characterised by the presence of the bacterium in the body without causing overt illness.^2,4–10^ However, asymptomatic infection harbours the potential to transition into active TB under certain circumstances, such as compromised immune defences or other coexisting factors. This dynamic ability of the host to move within the spectrum of disease adds an additional layer of complexity. Importantly, the dynamic is bi-directional: while it is intuitive that decreasing immune function increases the likelihood of disease, the inverse is also true: restoring immune function decreases risk of active disease, as dramatically demonstrated in HIV1-/MTB co-infection.

Among the intriguing yet understudied aspects of MTB infection is a phenomenon often referred to as the *mirage de tuberculose* in the literature. This enigmatic term encapsulates a scenario where individuals who possess detectable MTB, without apparent symptoms or radiological abnormalities. The detection is transient, meaning that the positive tests turn negative over time. Even while these individuals exhibit positive diagnostic tests or evidence of immune response, they defy the conventional clinical patterns associated with active TB disease.

The scientific understanding of the mirage de tuberculose remains significantly constrained.^11^ This limitation derives partly from the complex and challenging characteristics of the phenomenon. Ethical and public health considerations, as well as individual safety concerns, prevent the deliberate withholding of treatment from individuals with detectable MTB infections to observe spontaneous reversion to negative culture. Consequently, scientists are compelled to depend on serendipitous occurrences of the mirage de tuberculose as observed through inadvertent natural experiments.

The increasing challenge of multidrug-resistant TB (MDR-TB) has emerged as a pressing concern in the global fight against TB. MDR-TB refers to strains of MTB that are resistant to at least two of the most potent first-line anti-TB drugs, isoniazid and rifampicin. The global impact of MDR-TB is significant, with an estimated half a million cases occurring each year.^12,13^ It poses a threat to individual patients, as well as public health systems.

In this article, we describe a case of mirage de tuberculose with multidrug-resistant MTB, reviewed the literature and discussing challenges in individual patient management as well as the public health implications.

## CASE PRESENTATION

**FIGURE 1.**
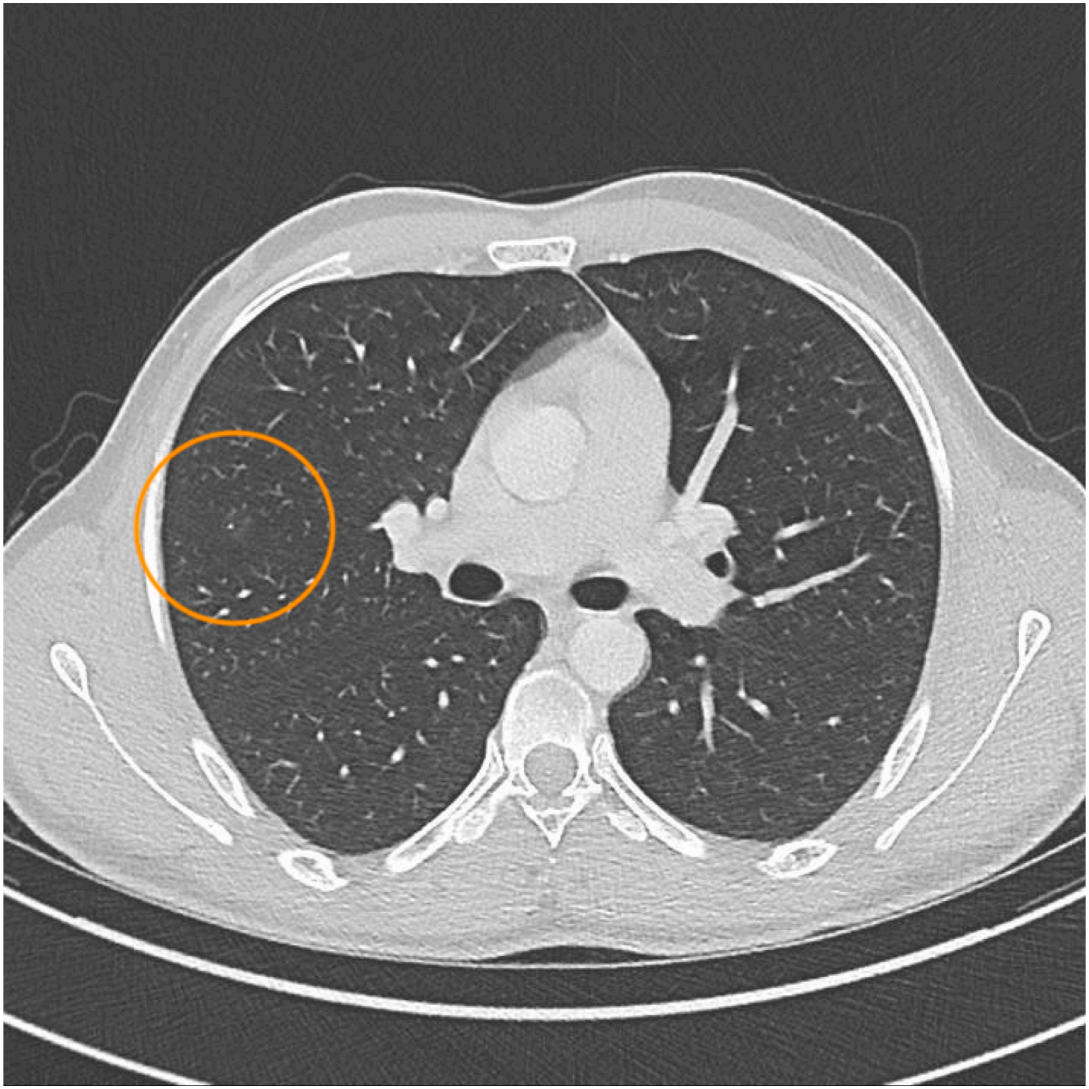
CT thorax at initial presentation showing small granuloma and surrounding ground glass opacity in the right middle lobe. CT = computed tomography.

**FIGURE 2.**
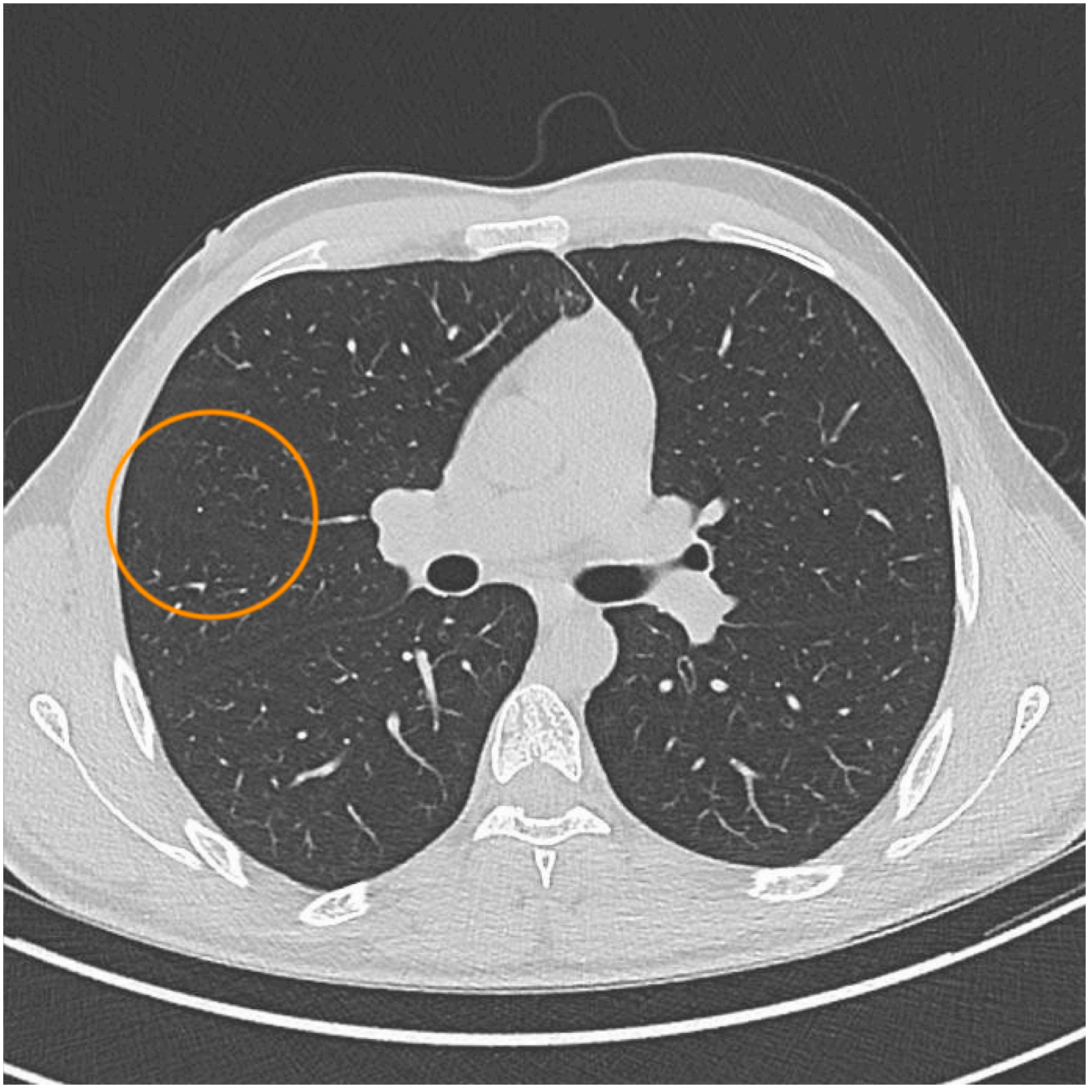
CT thorax at the time of treatment initiation showing persisting small granuloma and diminished ground glass opacities. CT = computed tomography.

A 23-year-old male patient exhibited a two-month history of night sweats, weight loss, and general weakness. Fever and respiratory symptoms were absent, and his medical history was unremarkable. The individual was a Somali refugee residing in Switzerland, having immigrated six years earlier after escaping as a minor through several countries including Ethiopia, Sudan, and Libya. Physical examination revealed no significant findings, and inflammatory markers (C-reactive protein [CRP], leucocytes) were within normal ranges. HIV testing yielded a negative result. A thoracic CT scan unveiled a small granuloma and subtle ground glass opacities in the right middle lobe (Figure 1). Initial sputum testing showed no acid-fast bacilli, and polymerase chain reaction (PCR) did not detect MTB DNA. However, eight weeks later, culture revealed growth of MTB, accompanied by resistance to rifampicin and isoniazid, indicative of MDR-TB. Molecular analysis from the MTB culture verified *rpo*B (S450L) and *kat*G (S315T) mutation. In addition, mutations in the *emb*B (M306I), *pnc*A (W68C) and *tly*A (N236K) gene were detected that are associated with ethambutol, pyrazinamide and capreomycin resistance. This unique pattern of resistances and resistance mutations assigned the MTB isolate to the European MDR-TB cluster among refugees from the Horn of Africa in 2016, which was confirmed by typing with next generation sequencing.^14^ During that period, the patient reported ongoing night sweats, while his weight remained stable. While again one out of two initial sputum samples showed culture positivity for MTB at the time of MDR-TB diagnosis, acid-fast bacilli and PCR remained persistently negative. CT scan upon MDR-TB diagnosis demonstrated diminishing ground glass opacities, with the small granuloma being the only persistent feature (Figure 2). All sputum samples collected were judged to be of adequate quality, and they underwent culturing using the BACTEC™ MGIT™ 960 system (BD, Franklin Lakes, NJ, USA). To eliminate the possibility of cross-contamination in the laboratory, genetic confirmation of MDR-TB was conducted on the MTB cultures derived from both positive specimens.

Considering the potential risk of transmission, the patient was managed as though he had active TB. He responded well to the treatment regimen and exhibited good tolerability to the drugs. An MDR-TB treatment regimen with bedaquiline (6 months), linezolid, moxifloxacin, clofazimine, cycloserin together with pyridoxin for 12 months was established. His recovery was successful, and the patient's overall condition improved. In total, the patient gained 8 kg over the course of the therapy and the subsequent 6 months follow-up period. Table 1 encompasses a comprehensive overview of recorded symptoms, laboratory markers, microbiology samples, and radiological tests conducted.

**TABLE 1.** Characteristics of study cases.

	Initial presentation	At diagnosis	After treatment*	6-month follow-up
Symptoms	2 months history of night sweat, weight loss, general weakness	Persistent night sweat, stable weight	Weight gain of 3 kg	Further weight gain (+5 kg)
Laboratory markers	CRP, leucocytes within normal range	CRP, leucocytes within normal range		
Microbiology (sputum)	1/2 MTB culture-positive (after 2 months), direct microscopy-negative, PCR-negative	1/2 MTB culture-positive (after 1 month), direct microscopy-negative, PCR-negative	M1–M3: direct microscopy-negative, culture-negative, PCR not performed	
Radiology (CT scan)	Small granuloma and surrounding ground glass opacity in the right middle lobe	Persisting small granuloma and diminished ground glass opacities		

* Following 12 months of bedaquiline, linezolid, moxifloxacin, clofazimine, cycloserin.

CRP = C-reactive protein; MTB = *M. tuberculosis*; PCR = polymerase chain reaction; M = month; CT = computed tomography.

Informed consent for the case presentation was provided.

## NARRATIVE REVIEW OF PREVIOUS CLINICAL REPORTS

We aimed to perform a systematic review to understand the current state of knowledge about mirage de tuberculose. We defined mirage as a patient who has MTB detectable either by culture or other methods, no or minimal signs of disease and who reverts positivity without tuberculostatic drugs. We included only clinical cohorts, reports or studies with >5 patients. Reviews were excluded. We performed a literature search for ‘mirage de tuberculose’ and ‘mirage tuberculose’ in PUBMED, Embase and Google Scholar. This search yielded a total of five papers, of which one was relevant (a commentary from Reves et al.^11^ on a paper published by Lewis et al.^15^). Next, we screened the references from the commentary and found the original paper from Kent et al., who references two more papers. One of those, Gad et al.,^16^ fulfils the criteria for inclusion into our analysis. Overall, the literature is too heterogenous to perform a systematic analysis. Therefore, we focused on a narrative review of three different papers reporting on cohorts of patients published in 1937, 1967 and 2009 (Table 2).

**TABLE 2.** Details of existing literature on *mirage de tuberculose.*

First author	Publication year	Patient population	Culture-positive *n*	Transiently positive *n* (%)
Gad et al.^16^	1937	Hospitalised patients	17	10 (59)
Kent et al.^17^	1967	Military personnel	17	6 (35)
Reves et al.^11^	2009	Gold miners	25	6 (24)

The earliest paper we found was published in 1937 and reports from the Oresund Hospital in Copenhagen.^16^ Gad et al. describe on various groups of patients with suspected TB and the diagnostic yield of gastric lavage of mycobacterial culture. They report progression to active disease in seven patients out of 17 who were initially culture-positive in gastric lavage. This suggest that approximately 60% of patients who were initially culture-positive were mirage de tuberculose.

The second paper was published in 1967 by Kent et al. reporting on an MTB outbreak on a military ship.^17^ They found 42 recent MTB converters which were culture-positive but asymptomatic with a normal chest X-ray. The paper provides some follow-up on 17 of the culture-positive patients reporting that six were culture-positive only once, suggesting that approx. 35% of these recent converters had mirage de tuberculose.

Finally, in 2009, Lewis et al. reported on an active TB screening programme amongst South African goldminers.^15^ Of 25 asymptomatic individuals with a normal chest X-ray, six had transiently positive cultures, suggesting that 24% of these patients might have had mirage de tuberculose.

An important limitation in all these studies is the potential for sampling error or culture contamination to contribute to the observed mirage phenomenon. It is crucial to consider these factors when interpreting transient culture positivity for MTB. Despite stringent laboratory protocols, inadvertent contamination during specimen collection, transportation, or processing can result in false-positive culture results.

Sampling error, particularly in cases with intermittent shedding of MTB, may lead to inconsistent culture outcomes, further complicating the accurate characterisation of mirage de tuberculose cases. Additionally, variations in the quality and quantity of sputum samples collected from patients can influence the reliability of culture results, potentially introducing false-negative findings. Finally, the presence of non-tuberculous mycobacteria in sputum samples can also contribute to culture contamination and misinterpretation of results.

## HOW TO DIAGNOSE *MIRAGE DE TUBERCULOSE*

Diagnosing mirage de tuberculose proves to be exceptionally challenging due to the paucity of typical signs and symptoms associated with TB. While the advancement of modern radiological techniques might be presumed to offer heightened sensitivity compared to older methodologies, our case exemplifies that even with contemporary imaging tools, mirage de tuberculose remains essentially undetectable. Furthermore, non-cultural diagnostic approaches, such as PCR, proved ineffective in our case, further complicating the diagnostic process. Importantly, our experience underscores that only prolonged culture spanning several weeks ultimately unveiled the true nature of the patient's condition. This implies that the pursuit of mirage de tuberculose mandates repeated and extended culture procedures, presenting substantial challenges at the population level. As such, comprehensive efforts in diagnosing this elusive condition require innovative strategies and heightened awareness.

## THE DIFFERENCE BETWEEN *MIRAGE DE TUBERCULOSE*, SUBCLINICAL TB, AND ACTIVE TB DISEASE

Therapeutic decisions are often contingent upon the establishment of arbitrary cutoffs within this spectrum, reflecting the need for clinical management. Paradoxically, this spectrum-based approach has led to a proliferation of diverse categorisations within the field, lacking robust clinical validation.^18^ Amidst this complexity, the concept of subclinical TB emerges as a notable classification, encompassing bacteriologically confirmed MTB cases characterised by minimal or absent symptoms.^19^ This classification circumvents the quandary regarding the biological significance of positive PCR signals.^20,21^ Remarkably, the identification of subclinical TB demonstrates an 89% detection rate through the simplest form of X-ray, highlighting its potential as a diagnostic tool.^19^ In contrast to this definition, the lesions identified in the CT scan of our described case were barely discernible to the untrained eye. The sole diagnostic method utilised for this case was the positive MTB culture, which notably displayed transient positivity. Overall, this finding supports the hypothesis that a TB mirage exists within the TB spectrum, representing a stage preceding significant tissue destruction akin to subclinical TB, thus eluding detection through chest X-ray imaging and standard screening methods. Notably, all patients falling under the classification of subclinical TB received treatment, since these were national screening programmes. This contrasts starkly with the mirage cohorts, where treatment was not given due to the inherent nature of the study. This divergence underscores a critical differentiation, suggesting that individuals within the mirage group are situated closer to a state of natural immunity. Such a discrepancy becomes particularly significant as it implies that patients identified as subclinical TB cases exhibit readily discernible tissue destruction, albeit with limited symptoms, necessitating medical intervention. In contrast, the untreated mirage cases, characterised by their absence of therapeutic intervention, potentially represent a stage of MTB infection that is more akin to an immune-regulated response without the necessity for medical interference.

**FIGURE 3.**
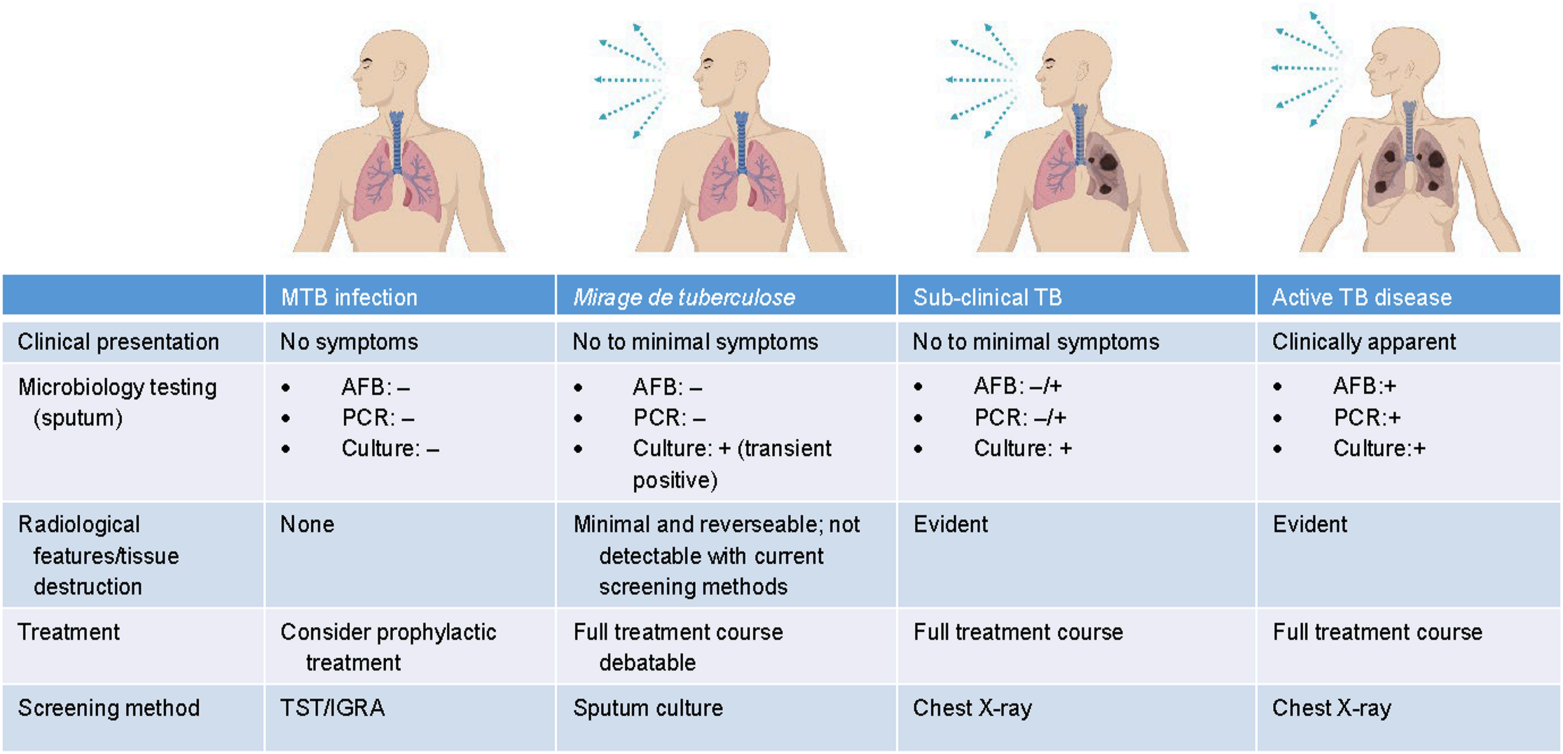
Spectrum of TB infection to active disease. MTB = *M. tuberculosis*; AFB = acid-fast bacilli; PCR = polymerase chain reaction; – = negative; + = positive; TST = tuberculin skin test; IGRA = interferon-gamma release assay.

Notably, HIV-positive patients and children also often exhibit no or minimal symptoms. However, this is not a result of immune control but rather the lack of an appropriate immune response. Therefore, these patient groups tend to have more disseminated disease and higher bacterial loads, representing a different end of the disease spectrum compared to “mirage de TB”. We summarised the spectrum of TB infection to TB disease, including our proposed features for mirage de tuberculose in Figure 3.

## HOW TO TREAT *MIRAGE DE TUBERCULOSE*

In the context of mirage de tuberculose, there are no established treatment recommendations. The cultural detection of MTB suggests a close resemblance to active TB. However, predicting the future course of infection or the potential for reactivation remains uncertain. This poses challenges in determining the appropriate treatment approach for these patients. Patients with mirage de tuberculose exhibit characteristics that resemble those who might qualify for shorter treatment durations.^22^ In this specific case, we opted to administer the shortest possible active treatment regimen available for our patient with mirage de tuberculose.

## PUBLIC HEALTH ASPECTS/INFECTION PREVENTION AND CONTROL

The detection of MTB in airway samples traditionally reflects infectivity. However, the extent of infectivity varies by diseases characteristics such as bacillary load, extent of lung involvement and symptoms. Absence of respiratory symptoms and cavitary lesions is likely associated with less spread of the disease.^23^ Infectious aerosols are generated predominantly by coughing.^24^ Given the unpredictable nature of disease (early active TB vs. mirage de tuberculose) and the potential risk of secondary cases, it seems prudent to initiate aerosol precautions and perform contact tracing according to guidelines until future studies provide a better estimate of infectiousness of these patients.^25^
